# Negative feedback regulation of the ERK1/2 MAPK pathway

**DOI:** 10.1007/s00018-016-2297-8

**Published:** 2016-06-24

**Authors:** David Lake, Sonia A. L. Corrêa, Jürgen Müller

**Affiliations:** 1 Warwick Medical School, University of Warwick, Coventry, UK; 2 School of Life Sciences, University of Warwick, Coventry, UK; 3 Faculty of Life Sciences, University of Bradford, Bradford, UK; 4 Aston Medical Research Institute, Aston Medical School, Aston University, Birmingham, B4 7ET UK

**Keywords:** Cell signalling, Negative feedback, Signalling dynamics, Spatiotemporal regulation, Pathway modelling, Cancer

## Abstract

The extracellular signal-regulated kinase 1/2 (ERK1/2) mitogen-activated protein kinase (MAPK) signalling pathway regulates many cellular functions, including proliferation, differentiation, and transformation. To reliably convert external stimuli into specific cellular responses and to adapt to environmental circumstances, the pathway must be integrated into the overall signalling activity of the cell. Multiple mechanisms have evolved to perform this role. In this review, we will focus on negative feedback mechanisms and examine how they shape ERK1/2 MAPK signalling. We will first discuss the extensive number of negative feedback loops targeting the different components of the ERK1/2 MAPK cascade, specifically the direct posttranslational modification of pathway components by downstream protein kinases and the induction of *de novo* gene synthesis of specific pathway inhibitors. We will then evaluate how negative feedback modulates the spatiotemporal signalling dynamics of the ERK1/2 pathway regarding signalling amplitude and duration as well as subcellular localisation. Aberrant ERK1/2 activation results in deregulated proliferation and malignant transformation in model systems and is commonly observed in human tumours. Inhibition of the ERK1/2 pathway thus represents an attractive target for the treatment of malignant tumours with increased ERK1/2 activity. We will, therefore, discuss the effect of ERK1/2 MAPK feedback regulation on cancer treatment and how it contributes to reduced clinical efficacy of therapeutic agents and the development of drug resistance.

## Introduction

### The ERK1/2 MAPK pathway

Eukaryotic cells respond to changes in their environment through complex and interconnected signal transduction networks that convert external stimuli into a range of cellular responses. A common motif by which extracellular modulation of cell-surface receptor activity is transduced into a specific cellular response is the three-tiered mitogen-activated protein kinase (MAPK) cascade, with the extracellular signal-regulated kinase 1/2 (ERK1/2) pathway being the most extensively investigated. The principal mechanisms involved in the activation of the ERK1/2 pathway have been well characterised [1]. In brief, signalling is initiated when an extracellular ligand binds to a specific receptor tyrosine kinase (RTK) at the plasma membrane. This promotes receptor dimerisation and autophosphorylation on intracellular tyrosine residues that then act as recognition sites for proteins containing Src homology 2 (SH2) or phosphotyrosine binding (PTB) domains, including the adaptor proteins Shc and Grb2. Son of sevenless (SOS) is then recruited from the cytosol to the plasma membrane through Shc and Grb2 and acts as the major guanine nucleotide exchange factor (GEF) that catalyses the conversion of inactive Ras-GDP to active Ras-GTP [2]. Activated Ras-GTP then recruits Raf to the plasma membrane, where it is activated in a complex fashion (for review, see [3, 4]). Once activated, all Raf family members (A-Raf, B-Raf, and c-Raf (Raf-1) [5]) are capable of activating MEK1/2, which in turn activates ERK1/2. This is achieved by phosphorylation in the activation segment of their respective kinase domains. The ultimate outcome is the phosphorylation of a large variety of critical targets by ERK1/2. Over 150 ERK1/2 substrates, including many nuclear transcription factors, have been identified [6]. The specific set of targets that are phosphorylated under particular extra- and intracellular conditions determines the appropriate response of the cell.

### Regulation of the ERK1/2 signalling pathway

To adapt to particular environmental circumstances, the ERK1/2 MAPK pathway must be integrated into the overall signalling activity of the cell. Importantly, the magnitude, duration, and location of MAPK signalling must be strictly controlled to produce the correct biological response. Multiple mechanisms have evolved to perform this role, including positive and negative feedback loops as well as extensive cross talk between parallel pathways. In this review, we will focus on negative feedback and the mechanisms by which it shapes ERK1/2 MAPK signalling. Negative feedback loops targeting the ERK1/2 MAPK cascade fall into two main categories: direct posttranslational modification of pathway components and the induction of *de novo* gene synthesis of specific pathway inhibitors. The principal difference between these two mechanisms is the time required to take effect. While direct posttranslational modification is nearly instantaneous, *de novo* gene expression and protein synthesis is somewhat delayed following the initial pathway activation. In this review, we will discuss in detail how negative feedback determines ERK1/2 spatiotemporal signalling dynamics and the role of negative feedback regulation in the development and treatment of cancer.

### Inhibitory feedback phosphorylation by downstream kinases

**Fig. 1:**
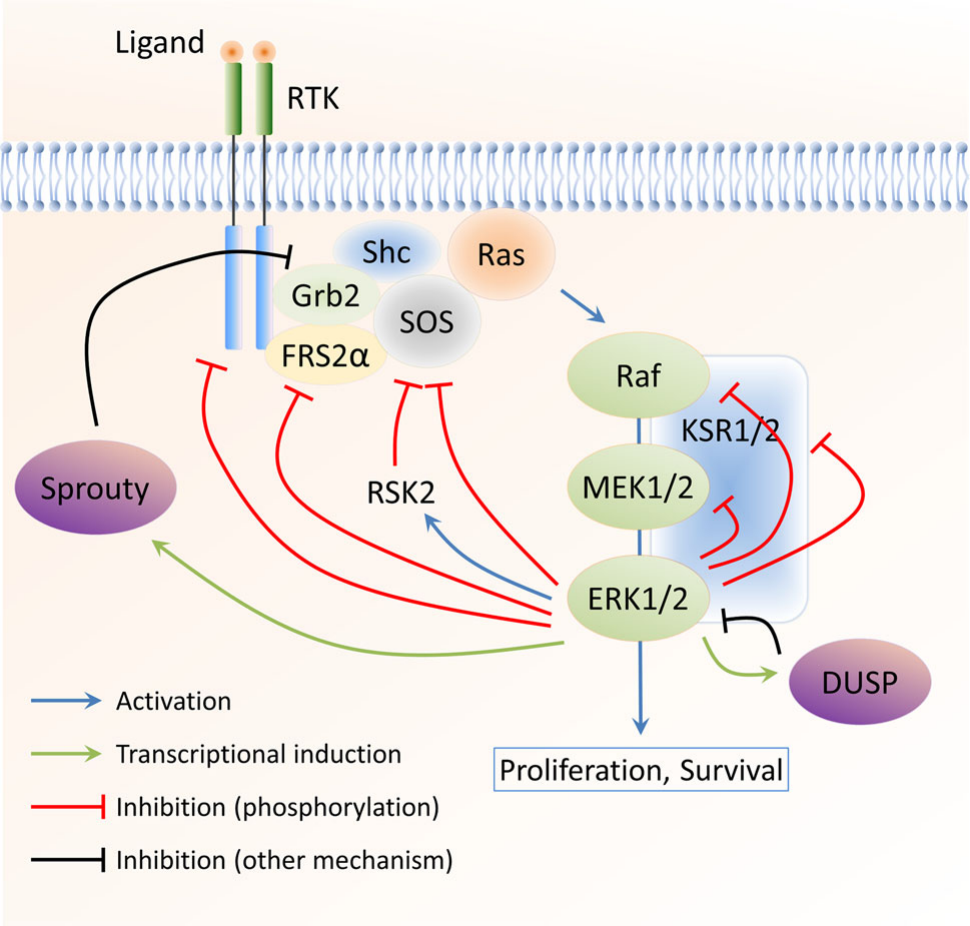
Negative feedback regulation of the ERK1/2 MAPK pathway. The ERK1/2 MAPK pathway is subject to a large number of negative feedback loops. These include direct phosphorylation by ERK1/2 (and RSK2) as well as transcriptionally induced feedback regulators, such as DUSPs and Sprouty proteins. The major negative feedback loops acting on the ERK1/2 pathway are shown

Nearly all components of the ERK1/2 MAPK cascade are regulated through negative feedback phosphorylation by downstream kinases. The negative feedback phosphorylation events that have been studied in reasonable detail are summarised in Table 1 and Fig. 1 and will be further discussed in this article.

**Table 1:** Negative feedback phosphorylation of ERK1/2 MAPK pathway components

Component	Phosphorylation sites	Mechanism	References
EGFR	T669	Reduces EGFR tyrosine phosphorylation through impaired cross activation of the receptor dimer	[7–11]
FGFR1	S777	Reduces FGFR1 tyrosine phosphorylation (unknown mechanism)	[14]
SOS1	S1132, S1167, S1178, S1193	Disrupts association with Grb2	[15–19]
SOS1	S1134, S1161 (phosphorylated by RSK2)	Creates a 14-3-3 binding site; reduces plasma membrane localisation	[20, 21]
FRS2α	T132, T135, T138, T376, T452, T455, T458, T463	Reduces FRS2α tyrosine phosphorylation; inhibits Grb2 association	[23, 24]
LAT	T155	May disrupt association with PLCγ1	[26]
Raf-1	S29, S289, S296, S301, S642	Reduces association with Ras and plasma membrane localisation	[15, 28–31, 33, 34]
B-Raf	S151, T401, S750, T753	Disrupts dimerisation with Raf-1 (S151, T401, S750, T753); disrupts association with Ras (S151)	[35, 36]
MEK1	T292, T386	May increase the dephosphorylation of the MEK1 activating sites; interferes with the activating phosphorylation of MEK1 on S298 by PAK (T292)	[38–41]
KSR1	T260, T274, S320, S443, S463	Disrupts association with B-Raf; regulates subcellular localisation (compartmentalisation)	[54–57]

The ERK1/2 pathway proteins known to be phosphorylated in a negative feedback loop are listed, together with the phosphorylated residue(s). All sites are directly phosphorylated by ERK1/2, unless specified otherwise. The potential mechanisms by which these modifications regulate the target protein are also listed

#### Growth factor receptors

ERK1/2 proteins have been reported to directly phosphorylate several receptor tyrosine kinases. For example, ERK1/2 MAPKs can phosphorylate the epidermal growth factor (EGF) receptor (EGFR) at T669 [7, 8], the major site of Ser/Thr phosphorylation of the receptor. Phosphorylation on this conserved residue, which is located in the juxtamembrane region of the receptor, was shown to be induced by tumour necrosis factor (TNF)-α, EGF, or heregulin (HRG) in a breast cancer cell line [9]. T669 phosphorylation was dependent on ERK1/2 activity and was subsequently shown to reduce the level of constitutive EGFR tyrosine phosphorylation, suggesting that it could downregulate EGFR signalling. The mechanism involved appears to be a reduced ability of the phosphorylated juxtamembrane region to cross activate the other receptor kinase of the dimer, even across heterodimeric receptors [9]. ERBB3 (erb-b2 receptor tyrosine kinase 3) has been shown to be phosphorylated in a position similar to T669 of the EGFR, leading to reduced tyrosine phosphorylation of the receptor [10], likely through a similar mechanism. Interestingly, another report demonstrated that while EGF-stimulated, ERK1/2-dependent phosphorylation of T669 reduced tyrosine phosphorylation and the activity of the EGF receptor, T669 phosphorylation also inhibited receptor downregulation, thereby acting as a positive regulator of signalling [11]. It is, therefore, possible that the same phosphorylation event controls both the duration of the initial signal as well as the subsequent internalisation of the receptor. An attractive hypothesis is that the negative regulation of EGFR activity by T669 phosphorylation is the event that causes the reduced internalisation, as receptor internalisation is significantly increased by EGFR activity [12]. However, exactly how these two outcomes influence the final dynamics of the pathway awaits further characterisation. Interestingly, ERK1/2 activity can also activate the tyrosine phosphatase cdc25c via phosphorylation at T48 [13]. Cdc25c then dephosphorylates the EGFR at Y1068, which is required for its activity, in a negative feedback loop. Therefore, in addition to phosphorylating the receptors directly, ERK1/2 can also activate tyrosine phosphatases that inactivate the EGFR, adding to the number of mechanisms by which upstream RTK signalling can be suppressed by negative feedback.

The fibroblast growth factor (FGF) receptor has also been shown to be subject to ERK1/2 feedback phosphorylation. FGF signalling leads to FGF receptor 1 (FGFR1) phosphorylation on a conserved serine residue (S777) in its C-terminal tail in an ERK1/2-dependent manner [14]. Furthermore, the authors demonstrated that recombinant ERK1 and ERK2 phosphorylate FGFR1 on S777 in vitro. Importantly, the mutation of S777 to alanine resulted in prolonged FGF1-induced tyrosine phosphorylation of FGFR1. It also increased cell proliferation and migration of U2OS cells expressing FGFR1, as well as neuronal outgrowth in cultured dorsal root ganglions, two established models of FGFR-stimulated, ERK1/2-dependent cell function. These experiments clearly demonstrate that FGFR1 is subject to direct negative feedback phosphorylation by ERK1/2. It is unclear, however, how this results in decreased FGFR1 tyrosine phosphorylation and attenuated signalling. It is possible that the phosphorylated S777 site acts as a binding site for tyrosine phosphatases, or that S777 phosphorylation induces local conformational changes in FGFR1 that make it a better substrate for dephosphorylation, but these possibilities have yet to be investigated.

#### Adaptor proteins, exchange factors, and GTP hydrolyase activating proteins

Adaptor proteins connect activated RTKs to Ras and the downstream MAPK cascade. Regardless of continuous growth factor stimulation, Ras activation is usually transient. A negative feedback loop from ERK1/2 to adaptor proteins and exchange factors contributes to this transiency. For example, it was found that MAPK pathway activation results in hyperphosphorylation of SOS1, which was enhanced by ERK overexpression [15] and prevented by MEK1/2 inhibition [16]. It was further shown that SOS1 is phosphorylated in vitro by activated ERK2 at multiple sites [17]. SOS1 phosphorylation disrupts its association with the adaptor proteins Grb2 and Shc and, consequently, the receptors at the plasma membrane [16–18]. Interestingly, simulation modelling suggests that the sites phosphorylated by ERK1/2 may act decisively—as opposed to cooperatively—in downregulating SOS1 activity [19]. SOS1 is also phosphorylated by the ERK1/2 effector ribosomal S6 kinase 2 (RSK2) [20]. RSK2-mediated phosphorylation of two sites on SOS1 (S1134 and S1161) leads to the recruitment of 14-3-3 and negative regulation of ERK1/2 activity [21].

The FGF receptor substrate 2 proteins (FRS2α and FRS2β) are adaptor proteins that are recruited to activated RTKs via N-terminal PTB domains. They also contain multiple tyrosine phosphorylation sites in their C-termini that allow the binding of the adaptor protein Grb2 [22]. Lax and colleagues [23] found that upon FGF stimulation FRS2α became phosphorylated on eight threonine residues, all of which were followed by a proline, representing a minimum ERK1/2 consensus sequence. They further showed that expression of dominant-negative Ras or MEK1/2 inhibition abolished this phosphorylation, suggesting that FRS2α is phosphorylated by an ERK1/2-dependent feedback mechanism. Importantly, mutation of the FRS2α threonine phosphorylation sites enhanced FRS2α tyrosine phosphorylation and Grb2 association. The same FRS2-dependent mechanism has been reported for the regulation of EGFR signalling [24], suggesting that threonine phosphorylation of FRS2α by ERK1/2 may represent a conserved mechanism by which to inhibit FRS2α tyrosine phosphorylation and downregulate RTK signalling.

Another adaptor protein regulated by ERK1/2 feedback phosphorylation is the linker for activation of T cells (LAT), a single-pass transmembrane protein that is expressed in various immune cells [25]. Upon engagement of the T cell receptor (TCR), tyrosine phosphorylation of LAT leads to the recruitment of several SH2 domain-containing proteins, including Grb2, resulting in ERK1/2 activation. Matsuda and colleagues [26] found that LAT can be phosphorylated on T155 by ERK1/2 in vitro. They further showed that this site is phosphorylated upon T cell activation in a MEK1/2-dependent manner, suggesting that this event represents a feedback mechanism. To this end, they showed that a LAT T155A mutant supported an enhanced degree of TCR signalling (as measured by an increase in intracellular Ca^2+^ mobilisation) and that abolishing ERK1/2 feedback through a MEK1/2 inhibitor similarly enhanced TCR signalling. Through co-immunoprecipitation experiments, the authors showed that the mechanism behind the inhibitory role of LAT T155 phosphorylation may involve the disruption of the interaction of LAT with its downstream signalling partner, phospholipase C γ1 (PLC γ1), but not Grb2.

A recent paper by Hennig et al. [27] also implicated Ras-GAP proteins as a target of negative feedback regulation of Ras activity. They showed that inhibition or knockdown of MEK1/2, ERK1/2, or RSK in HeLa cells resulted in sustained activity of Ras (evidenced by GTP loading). As the GDP/GTP exchange rate at the later time points was not significantly changed, they concluded that the sustained activity must be due to the inactivation of a Ras-GAP, rather than an exchange factor such as SOS. Through knockdown experiments, the authors then implicated NF1 (neurofibromin) as the likely candidate, as knockdown resulted in similarly sustained Ras activation kinetics as the inhibition of ERK1/2 or Rsk. While it remains to be shown whether NF1 activation by ERK1/2 and Rsk is by direct phosphorylation or if other proteins/mechanisms are involved, this work adds another route by which the upstream components of the ERK1/2 MAPK cascade can be inhibited through negative feedback.

#### Raf proteins

The first suggestion that Raf is subject to feedback phosphorylation was provided by Ueki and colleagues [15], who showed that Raf-1 is hyperphosphorylated after the stimulation of insulin receptor-expressing CHO cells with either insulin or TPA (12-*O*-tetradecanoylphorbol 13-acetate). Raf-1 hyperphosphorylation was delayed compared to the activation of Raf-1, MEK1/2 and ERK1/2, and it was further enhanced by overexpression of ERK1, suggesting a negative feedback loop. Further studies showed that Raf-1 hyperphosphorylation is dependent upon MEK1/2 activity [28] and that pharmacological inhibition of ERK1/2 signalling results in increased and more sustained Raf-1 activity compared to its normal transient activation [29, 30]. Hyperphosphorylated Raf-1 also displayed reduced plasma membrane association, indicating a possible mechanism of Raf-1 feedback regulation [28].

A major advancement towards understanding the role of Raf-1 in the negative feedback regulation of ERK1/2 signalling was made by the identification of six Raf-1 residues that are phosphorylated following mitogenic stimulation [31]. Five of these sites were directly phosphorylated by ERK2 in vitro, and their phosphorylation was dependent on signalling downstream of MEK1/2, suggesting that they are involved in a feedback loop. The identified phosphorylation sites are located close to the N-terminus (S29/S43), the C-terminus (S642), and in the flexible hinge region between the regulatory and catalytic domain (S289/S296/S301) of Raf-1. Phosphorylation at these sites yielded a desensitised Raf-1 unable to localise to the plasma membrane and to engage with activated Ras [31]. Feedback phosphorylation also prevented sustained Raf-1 activity [31, 32], as was shown by MEK1/2 inhibition as well as mutation of the feedback sites. In addition, desensitised (hyperphosphorylated) Raf-1 cannot be activated by stimulation with another growth factor, even though the upstream Ras protein is activated. Interestingly, hyperphosphorylated Raf-1 is not degraded, but is resensitised by a complex process involving the prolyl isomerase Pin1 and the protein phosphatase PP2A [31]. ERK1/2 activity-dependent phosphorylation of a subset of these sites (S296/S301) has also been observed by Hekman et al. [33], following expression of an activated Raf-1 mutant in Drosophila Sf9 cells. Importantly, these authors showed that the mutant proteins had significantly increased kinase activity in quiescent and EGF-stimulated mammalian cells, confirming a negative regulatory role for this phosphorylation event. However, while Balan et al. [34] also described phosphorylation of a subset of these sites (S289/S296/S301) in response to EGF stimulation of COS-7 cells, they found that it rather acts in a stimulatory manner. The reasons for this discrepancy are not clear, but could involve the different cell lines (NIH3T3 vs. COS-7) or growth factors [Platelet-derived growth factor (PDGF) vs. EGF] used. In addition, the ERK1/2 feedback sites located at the N- and C-terminus of Raf-1 (S29 and S642, respectively) were not characterised by Balan et al. It is possible that phosphorylation of the different sites is hierarchical, with some sites being more important than others. It has also not been clarified if phosphorylation at the multiple sites of Raf-1 is cooperative or decisive, i.e., whether all sites need to be phosphorylated for a measurable effect on Raf-1 activity. An interesting hypothesis is that Raf-1 feedback phosphorylation could serve as a sensor for the strength of signalling output, responding differently to weak or strong Raf-1 activation. To gain more insight into these questions, the relative contribution of each of the identified ERK1/2 feedback sites to the overall regulation of Raf-1 activity clearly needs further investigation.

Feedback phosphorylation at ERK1/2 phosphorylation motifs was also identified within the B-Raf C-terminus (S750 and T753) upon engagement of the B cell antigen receptor in chicken DT40B cells [35]. Mutation of these sites to a phosphomimetic amino acid strongly reduced the ability of B-Raf to induce neurite outgrowth in a PC12 cell system. Interestingly, the S750 site is located in a similar sequence and position to one of the Raf-1 feedback phosphorylation sites (S642) [31]. It has since been shown that two additional sites within B-Raf (S151, T401) are phosphorylated by activated ERK1/2 in response to PDGF treatment of NIH3T3 cells [36]. These sites are located in the N-terminal region of B-Raf (in an equivalent position to S29 of Raf-1) and the flexible hinge region (similar to S289/S296/S301 of Raf-1), respectively. Together, these sites represent an arrangement similar to the feedback sites of Raf-1. They are also subject to Pin1/PP2A-dependent dephosphorylation, which may recycle it to a signalling-competent state. B-Raf had previously been found to heterodimerise with Raf-1 upon activation by Ras, leading to Raf-1 transactivation [37]. Mutational analysis suggested that ERK1/2 phosphorylation of all four sites within B-Raf contributes to disruption of its dimerisation with Raf-1, whereas phosphorylation at S151 is solely responsible for inhibiting the interaction with activated Ras [36]. Interestingly, the combined mutant had a stronger oncogenic effect in focus formation assays than the single mutants (other combinations have not been investigated), indicating a degree of additivity.

#### MEK1/2

A number of inhibitory, proline-directed phosphorylation sites have been reported for MEK1, including T286, T292, and T386 [38–41]. The T292 site has been described in each of these studies and appears to play the decisive role in inhibiting MEK1 activity. T292 has been shown to be phosphorylated by ERK1 and ERK2 in vitro and its phosphorylation is dependent on ERK1/2 activity in intact cells [38–40]. Phosphorylation of T292 inhibits the in vitro kinase activity of MEK1 towards ERK1/2 [41], and the T292A mutant is activated more strongly by serum treatment [38], indicating a negative regulatory role for T292 phosphorylation. Dephosphorylation and inactivation of MEK1 (and MEK2) has been proposed as a potential mechanism mediating this feedback. Eblen and colleagues [40] further found that ERK1/2 phosphorylation of MEK1 on T292 interferes with the binding of MEK1 to ERK2. It also reduced the ability of PAK (p21-activated kinase) to phosphorylate MEK1 on S298, which is required for the activation of MEK1 by cell adhesion [39, 40, 42]. Interestingly, the equivalent of the T292 site is absent in MEK2. Nevertheless, it has been shown that MEK1 and MEK2 form heterodimers in vivo and that phosphorylation of T292 in MEK1 also reduces the activity of MEK2 in the context of the dimer [43], thus enabling the two MEK proteins to be regulated simultaneously.

#### KSR1

A number of scaffold proteins have been shown to regulate MAPK signalling in mammalian cells by organising the signalling components into macromolecular complexes and enhancing the efficiency and specificity of the MAPK signalling cascade [44–46]. KSR (kinase suppressor of Ras) proteins are perhaps the best studied scaffold proteins for the ERK1/2 MAPK pathway. KSR1 was originally identified as a positive regulator of Ras signalling in *Drosophila* and *C. elegans* [47–49]. Subsequent analysis found that KSR1 interacts with all three kinases of the ERK1/2 cascade [50, 51]. KSR1 has also been shown to translocate from the cytosol to the plasma membrane upon growth factor stimulation [52, 53], thus allowing the assembly of the ERK1/2 pathway (Raf, MEK1/2, and ERK1/2) close to the upstream activators (i.e., activated Ras). Importantly, KSR1 translocation also localises active ERK1/2 into close proximity to Raf-1 and other pathway constituents, potentially facilitating feedback phosphorylation of upstream pathway components.

A number of KSR1 residues (T256, T260, T274, S320, S443, S463) corresponding to the minimum ERK1/2 phosphorylation consensus motif (SP or TP) have been shown to be phosphorylated in cycling cells [54] as well as those stimulated with active Ras or growth factors [55–57]. These sites were also phosphorylated by recombinant ERK1/2 in vitro and their phosphorylation in intact cells depended on MEK1/2 activity [55–57]. KSR1 was also phosphorylated at a subset of these sites when it was immunoprecipitated from cycling HEK293T cells [54], indicating that the responsible kinase(s) associated with KSR1. As activated ERK1/2 associates with KSR1 in a Ras-dependent manner [55], this further suggests that ERK1/2 may directly phosphorylate KSR1.

Phosphorylation of KSR1 at the above sites has been shown to have multiple effects on its function. Mutation of these sites, inhibition of MEK1/2, and blocking ERK1/2 binding to KSR1 all resulted in increased and sustained binding of KSR1 to B-Raf, suggesting that KSR1 feedback phosphorylation interrupts the ternary complex of B-Raf, KSR1, and MEK1/2 [57]. Subsequent co-immunoprecipitation experiments demonstrated that ERK1/2 feedback phosphorylation of KSR1 leads to the release of KSR1 from the plasma membrane, thereby impairing the ability of KSR1 to potentiate signal transduction. KSR1 feedback phosphorylation has also been shown to influence signalling dynamics, as KSR1 feedback mutants show sustained ERK1/2 activation in response to EGF treatment of HEK293 cells [56]. Interestingly, the S443 site has been shown to be the most critical feedback site [56], as mutation of this residue alone resulted in significantly prolonged ERK1/2 activation in HEK293 cells, while further mutation of other sites (T260, T274, and S320) had a small additive effect.

The molecular mechanisms by which feedback phosphorylation of KSR1 regulate signalling were further analysed in detail in neurons [56], where KSR1 is most strongly expressed [58, 59]. ERK1/2 activity is well known to be required for synaptic plasticity, particularly long-term potentiation (LTP) of synaptic currents, by regulating the amount of glutamate receptors at the cell surface of the postsynaptic compartment, the dendritic spines. Notably, KSR1 has been demonstrated to be located in dendritic spines [56], suggesting for the first time that it may regulate synaptic strength by promoting local ERK1/2 activity in the postsynaptic compartment. Furthermore, it was demonstrated that feedback phosphorylation of KSR1 results in the exclusion of the ERK1/2 scaffold complex from dendritic spines, thus reducing ERK1/2 activity specifically in the postsynaptic compartment. As postsynaptic ERK1/2 activity is essential to promote LTP, the localised decrease of ERK1/2 activity had a significant impact on synaptic plasticity, as shown by electrophysiological experiments [56]. Overall, this study demonstrated that in this system, negative feedback limits compartmentalised signalling output to ensure that it remains within physiologically relevant boundaries.

#### Summary

Almost every step in the ERK1/2 MAPK pathway is targeted by negative feedback phosphorylation (Fig. 1), from the growth factor receptors at the plasma membrane to the core components of the cascade (such as Raf-1/B-Raf and MEK1/2) and scaffold proteins, such as KSR1. Of those, the Raf proteins and KSR1 demonstrate complex regulatory mechanisms, are able to interact with several pathway components, and reversibly localise to different subcellular compartments. These two proteins may, therefore, serve as key regulatory elements of the ERK1/2 cascade that can finely tune the dynamics of ERK1/2 signalling in the cell as a whole as well as in specific subcellular compartments.

### Transcriptional induction of specific MAPK pathway inhibitors

In addition to rapid feedback by direct phosphorylation using pre-existing protein kinases, transcriptionally induced mechanisms of feedback control have been described for the ERK1/2 MAPK pathway. These processes are likely to take effect with some delay compared to direct phosphorylation. As such, they would be well suited to modulating the later phases of ERK1/2 signalling dynamics, rather than leading to the rapid termination of signalling. They would also be able to contribute to adjusting steady-state signalling levels under conditions of sustained pathway input. The two major groups of proteins that have been demonstrated to perform this function are discussed below.

#### MAPK phosphatases

ERK1/2 require phosphorylation of both threonine and tyrosine residues in the activation loop for full kinase activity. Type 1/2 Ser/Thr phosphatases, protein tyrosine phosphatases, and dual-specificity Thr/Tyr phosphatases have all been shown to dephosphorylate and inactivate the ERK1/2 proteins [60]. The largest and best studied group of phosphatases that specifically regulate MAPK activity are the dual-specificity MAPK phosphatases (MKPs; reviewed in [61]), a subgroup of the dual-specificity phosphatases (DUSPs). There are ten MKPs in mammalian cells, which can be divided into three groups. The first group comprises the nuclear proteins DUSP1/MKP-1, DUSP2, DUSP4/MKP-2, and DUSP5, which dephosphorylate ERK1/2, p38, and JNK (DUSP5 is ERK1/2-specific). The second group includes the cytoplasmically located ERK1/2-specific phosphatases DUSP6/MKP-3, DUSP7/MKP-X, and DUSP9/MKP-4, while the final group contains the p38/JNK-specific phosphatases DUSP8 (M3/6), DUSP10/MKP-5, and DUSP16/MKP-7. In addition, a number of atypical DUSPs have been identified. However, atypical DUSPs do not possess a MAPK-binding motif, and their role in the regulation of MAPKs is less clear.

Many members of the DUSP family are immediate early or delayed early genes that can be transcriptionally induced by ERK1/2 MAPK pathway activation [62]. While the degree and the dynamics of induction of the individual DUSPs vary depending on the cell type and the exact stimulus, their inducibility enables these proteins to play an important role in feedback regulation of ERK1/2 activity. For example, in NIH3T3 cells, DUSP6 expression is induced by FGF [63]. This induction is blocked by MEK1/2 inhibition, suggesting that DUSP6 may be involved in pathway feedback. Further analysis demonstrated that upregulation of DUSP6 is mediated by direct binding of the ERK1/2-responsive transcription factor ETS1 to the DUSP6 gene promoter [63, 64]. Overexpression of wild-type DUSP6 reduced the levels of EGF-stimulated phospho-ERK1/2 after two hours of stimulation, but a phosphatase-dead mutant DUSP6 had no effect [64]. These results indicate that ERK1/2-induced DUSP6 expression leads to feedback inhibition via dephosphorylation of ERK1/2. In addition, due to their restricted distribution, DUSPs can anchor inactive ERK1/2 in the nucleus or the cytoplasm [65, 66], potentially delaying their re-activation. It should be noted, however, that DUSPs only act on ERK1/2 and do not regulate the upstream components of the pathway.

#### Sprouty proteins

Another group of relatively well-characterised transcriptionally induced inhibitors of ERK1/2 signalling are the sprouty (Spry) proteins [67], originally identified in *Drosophila* as an inhibitor of Ras signalling downstream of various RTKs [68–70]. In mammals, the expression of all four Spry proteins (Spry1-4) can be induced by growth factor signalling [71, 72]. In particular, Spry2 mRNA expression is induced by various FGF members in human ovarian granulosa lutein cells, murine osteoblastic cells, bovine ovarian granulosa cells, and murine pancreatic buds [73–76]. In several cases, the RTK-mediated induction of Spry expression was abrogated by MEK1/2 inhibition, suggesting that the expression of the Spry proteins depends on ERK1/2 activity and that they may function as feedback modulators [74–76]. Interactions between Spry proteins and several ERK1/2 pathway components have been reported, although how these interactions may allow Spry to modulate signalling remains largely unclear [77]. Nevertheless, one group showed that Spry1 and Spry2 become phosphorylated on a conserved N-terminal tyrosine residue (Y53 or Y55, respectively) upon EGF or FGF stimulation of C2C12 cells [78]. Phosphorylation at this site creates a docking site for the SH2 domain of Grb2, which leads to the disruption of the association between Grb2 and the FGFR adaptor FRS2. This result suggested that Spry may inhibit signalling upstream of Ras, but other reports indicate that Spry may also inhibit the pathway downstream of Ras, possibly by binding to Raf-1 [79, 80]. Other mechanisms have been reported, demonstrating that the regulation of ERK1/2 signalling by the Spry proteins is complex and likely depends on cell type and the nature of the stimulus (see [81] for a detailed discussion). Taken together, current data suggest that Spry proteins can be induced by ERK1/2 pathway activation and that they inhibit ERK1/2 signalling by acting at multiple nodes in the pathway in a context-specific manner.

#### Summary

A multitude of mechanisms has developed to inhibit ERK1/2 MAPK signalling by negative feedback regulation (Fig. 1). These include relatively fast-acting mechanisms that utilise the downstream components of the cascade (mostly ERK1/2, but also RSK2) to directly modify the activity of various upstream components. In addition, mechanisms have evolved that depend on the *de novo* expression of proteins, which in turn target the ERK1/2 pathway at multiple levels. These mechanisms are likely to be slower, affecting the later phases of ERK1/2 signalling. This multitude of mechanisms ensures that ERK1/2 signalling dynamics can be controlled in a well-defined manner and that it can be adapted to the particular circumstances of individual tissues and environmental conditions.

### Negative feedback modulates ERK1/2 MAPK signalling dynamics

#### Temporal regulation of ERK1/2 signalling by negative feedback

ERK1/2 MAPK pathway output is determined by the integration of positive input (e.g., growth factors) and positive and negative regulatory events, such as cross-talk and feedback mechanisms. These events determine ERK1/2 signalling strength and duration and critically influence the physiological outcomes. In a classic study, Traverse and colleagues reported that in rat PC-12 cells, stimulation with nerve growth factor (NGF) or EGF resulted in neuronal differentiation or proliferation, respectively [82]. These strikingly different responses were linked to differences in the temporal dynamics of ERK1/2 activation, whereby NGF caused sustained ERK1/2 activation and nuclear translocation, while EGF induced a transient response without nuclear translocation of activated ERK1/2. Subsequent computer simulations suggested that negative feedback is the major determinant of ERK1/2 dynamics in this system [83]. This analysis was further supported by Santos and colleagues [84] using modular response analysis. Feeding data generated from pathway perturbations by RNA interference into a sensitivity analysis produced evidence of a negative feedback loop from ERK1/2 to Raf-1 when the pathway was stimulated with EGF, but a positive loop when stimulated with NGF. These findings were substantiated by the demonstration that through rewiring the relevant feedback systems using pharmacological compounds and inhibitors, the dynamics of ERK1/2 activation and cell fate could be reversed in each of the NGF- or EGF-stimulated states [84]. Interestingly, differential expression of scaffold proteins, such as KSR1, can also significantly modulate ERK1/2 dynamics in PC-12 cells. Simply increasing the concentration of the KSR1 scaffold has been shown to convert the temporary ERK1/2 signal resulting from EGF stimulation into a sustained one and to change cellular outcome from proliferation to differentiation [58]. Interestingly, ERK1/2 signalling dynamics have a similar, albeit reverse effect on adipogenesis [85]. Low concentrations of KSR1 promote ERK1/2 activation, C/EBPβ phosphorylation, and adipogenesis. In contrast, higher concentrations of KSR1 were shown to prolong ERK1/2 signalling [86], promote PPARγ phosphorylation and inactivation, and inhibit differentiation (adipogenesis) in favour of proliferation [85, 86]. Together, these reports clearly highlight the power of feedback mechanisms to shape MAPK signalling outputs and the resulting physiological outcomes and suggest the existence of growth factor-specific network topologies.

Subsequent modelling approaches have further illuminated the role of negative feedback in determining ERK1/2 signalling outputs. Elegant combinatorial work by Sturm and colleagues [87] showed that the ERK1/2 MAPK pathway exhibits properties of a negative feedback amplifier (NFA). Importantly, the presence of negative feedback confers graded response characteristics (as opposed to a switch-like pattern when negative feedback is broken), robustness to change and stabilisation of output. The robustness conferred by negative feedback was further demonstrated in experiments showing that the level of steady-state doubly-phosphorylated ERK1/2 (‘output’) was only weakly dependent on ERK1/2 protein concentration [88], ensuring a reliable interpretation of extracellular signals under fluctuating conditions. The authors further showed that this robustness was lost in cells expressing constitutively active B-Raf mutants or a constitutively active Raf-1 protein, but not Ras mutants. As constitutively active B-Raf (or Raf-1) is not sensitive to negative feedback regulation, these experiments suggest that the robustness of the pathway output is critically dependent on negative feedback targeting Raf-1 [31].

#### Spatial regulation of ERK1/2 signalling by negative feedback

Many cellular processes occur in specialised subcellular compartments, and the localisation of ERK1/2 activity to those specific areas is critical to cellular function [1]. Modulating the subcellular localisation of activated ERK1/2, even without changing overall cellular ERK1/2 activity, can significantly change the availability of ERK1/2 for a particular molecular function, with important consequences for the outcome of signalling. Several cases have been described where feedback phosphorylation leads to the disassembly of the signalling complexes, resulting in the removal of components from the plasma membrane and termination of signalling [16–18, 31]. In addition, reversible spatial segregation of ERK1/2 pathway components by negative feedback has been demonstrated [56]. As described earlier, the scaffold protein KSR1 is subject to feedback phosphorylation by ERK1/2 at multiple sites. KSR1 is highly expressed in neurons [58], where it is involved in LTP (long-term potentiation), a process important for learning and memory [59]. Its localisation in primary hippocampal neurons was, therefore, further investigated. It emerged that mutant KSR1 that is unable to undergo feedback phosphorylation was preferentially present in dendritic spines of the neurons, where it colocalised with activated ERK1/2. In contrast, hyperphosphorylated KSR1 was largely absent from dendritic spines and resided in the dendritic processes. Importantly, MEK1/2 inhibition or reduction of neuronal activity by tetrodotoxin (TTX) resulted in the relocalisation of wild-type KSR1 to dendritic spines, demonstrating that the localisation of the KSR1 scaffold is reversibly regulated by neuronal activity and ERK1/2-dependent negative feedback. Localised ERK1/2 signalling in dendritic spines has been shown to be important for LTP and memory retention [89]. Consequently, the expression of a mutant KSR1 lacking ERK1/2 feedback phosphorylation sites in cultured hippocampal neurons led to increased KSR1 concentrations and phospho-ERK1/2 staining in dendritic spines and prolonged LTP of synaptic currents compared to wild-type KSR1. A model was proposed in which KSR1 tightly regulates MAPK cascade output in the postsynaptic compartment (dendritic spines) via compartmentalisation of the ERK1/2 signalling module. In resting neurons, KSR1 is localised to the postsynaptic compartment, where it scaffolds Raf, MEK1/2, and ERK1/2, making the synapse highly sensitive to Ras-activating signals that promote LTP. However, as more ERK1/2 is activated, feedback phosphorylation of KSR1 gradually removes it from the dendritic spines, making the system less sensitive and protecting against excessive ERK1/2 activity in the postsynaptic compartment. These results clearly demonstrate that negative feedback is able to direct the signalling complexes to different subcellular compartments, in addition to regulating their assembly and disassembly.

### Negative feedback regulation of ERK1/2 signalling in cancer progression and treatment

Aberrant ERK1/2 activation results in deregulated proliferation and malignant transformation in model systems and is commonly observed in human tumours. Inhibition of the ERK1/2 pathway, therefore, represents an attractive target for the treatment of malignant tumours with increased ERK1/2 activity. However, therapeutic agents targeting the ERK1/2 pathway would be expected to also inhibit the substantial negative feedback loops (see Fig. 1), with important consequences for therapeutic response and the development of drug resistance.

#### Sensitivity to negative feedback determines the response to therapeutic inhibitors

Activation of the ERK1/2 pathway is typically a result of mutations in members of the RAS and RAF gene families or the amplification and hyperactivation of RTKs [90–93]. Importantly, oncogenic B-Raf mutations are much more common than those in A-Raf or Raf-1. While A-Raf and Raf-1 require phosphorylation of two regions within their kinase domain for full activation [3], B-Raf contains two phosphomimetic aspartic acid residues and a constitutively phosphorylated serine at the equivalent positions [94]. Therefore, B-Raf has higher basal activity and requires only a single mutation within its kinase domain to switch to constitutively high activity. Such substitutions are frequently observed in ERK1/2-dependent tumours, most commonly the V600E mutation. Interestingly, tumours with B-RAF mutation are sensitive to inhibition of MEK1/2, whereas tumours with hyperactivated growth factor receptors are not [95]. Reasons for this discrepancy and potential ways to overcome these challenges are discussed below.

**Fig. 2:**
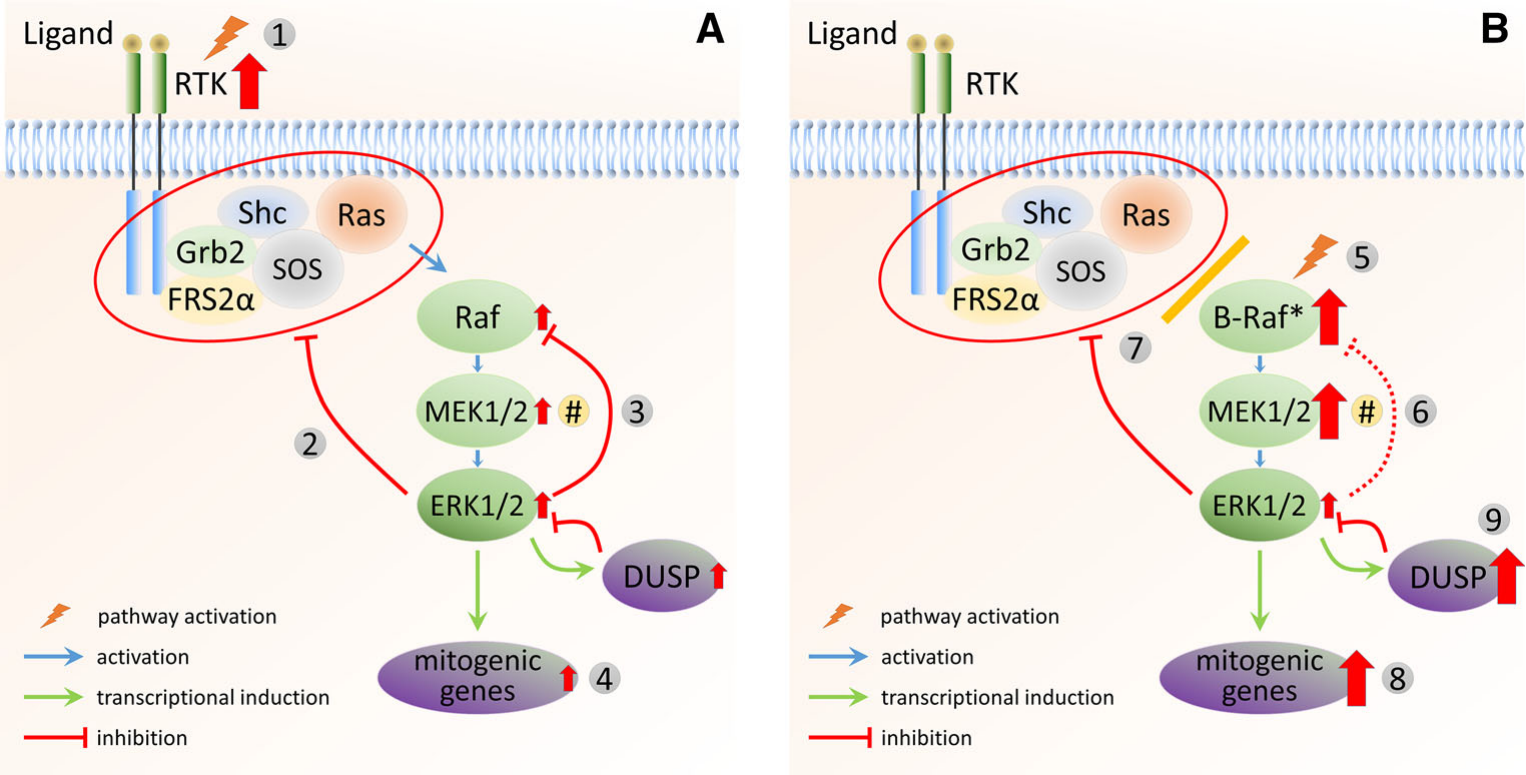
ERK1/2 MAPK signalling in response to different oncogenic stimuli. **a** In cells with mutation or amplification of upstream components [e.g., RTKs (*1*)] and expressing wild-type Raf proteins, negative feedback mechanisms are highly active (*2*,*3*) and significantly reduce the activity of several upstream pathway components. This leads to relatively low (but still elevated) steady-state levels of MEK1/2 and ERK1/2 activity. When either Raf or MEK1/2 are inhibited, this negative feedback is reduced. As a result, signal flux is increased, restoring MEK1/2 and ERK1/2 activity and requiring significantly higher inhibitor doses (intrinsic resistance). Loss of negative feedback (*2*) due to pathway inhibition also results in increased Ras-GTP levels, which promotes the dimerisation of wild-type Raf proteins and results in the paradoxical promotion, rather than inhibition, of ERK1/2 signalling. Finally, the observed transcriptional output (*4*) of tumour cells with mutated RTKs or Ras is only partially driven by ERK1/2 activity because of the relatively small increase in the overall signalling flux due to extensive negative feedback. Inhibition of Raf or MEK1/2, therefore, does not sufficiently reduce the expression of those mitogenic genes to result in therapeutic changes. **b** Mutant B-Raf (*5*) is constitutively active and, therefore, not sensitive to direct feedback phosphorylation by ERK1/2 (*6*). In addition, as mutated B-Raf is independent of upstream activation, negative feedback to the upstream components has no effect on B-Raf activity (*7*). Because mutated B-Raf bypasses negative feedback, persistent hyperactivation of MEK1/2 (and ERK1/2) results in significantly increased transcriptional output of mitogenic genes (*8*). As mitogenic gene expression critically depends on high signalling flux through the pathway, those tumours are sensitive to the inhibition of MEK1/2 or B-Raf. In addition, the increased expression of DUSPs (*9*) in B-Raf mutant cells leads to the dephosphorylation of ERK1/2 and a reduction of its apparent activity to levels that support oncogenic transformation (rather than senescence). As a result, MEK1/2 (rather than ERK1/2) activity is a major hallmark and determinant of inhibitor selectivity (*#*)

In cells with mutations in upstream components (e.g., RTKs or Ras) and expressing wild-type Raf proteins, negative feedback mechanisms significantly reduce the activity of several upstream pathway components, leading to relatively low (but still elevated) levels of MEK1/2 and ERK1/2 activity (Fig. 2a). Inhibition of MEK1/2 or Raf will weaken the negative feedback loops, leading to an increase in MEK1/2 and ERK1/2 activation. Therefore, a significantly higher inhibitor concentration is necessary for full inhibition of ERK1/2 signalling. This behaviour has been predicted by Sturm et al. [87], as loss of negative feedback is expected to increase the gain of the negative feedback amplifier (NFA) module, conferring robustness from external perturbations to the system. While this regulation helps to maintain signalling fidelity in normal cells, it also contributes to the development of intrinsic resistance to inhibitors. In addition, loss of negative feedback inhibition in these tumours results in increased levels of Ras-GTP, which has been shown to promote the dimerisation of Raf proteins [37, 96, 97]. As binding of a Raf inhibitor to one protomer can allosterically activate the other, Raf inhibition results in the paradoxical promotion, rather than inhibition, of ERK1/2 signalling [98, 99]. Importantly, the observed transcriptional output of tumour cells with mutated RTKs or Ras is only partially driven by ERK1/2 activity [100], with the activation of parallel pathways significantly contributing to the expression of mitogenic genes. In many cancers with upstream mutations, MEK1/2 or Raf inhibitors, therefore, do not result in therapeutic changes of gene expression. In this context, Sturm and colleagues [87] suggested that inhibiting targets outside of the NFA is likely to be more effective. Their modelling studies also raise the possibility of targeting modulators of ERK1/2 activity, such as scaffold proteins, either alone or in combination with Raf or MEK1/2 inhibitors. A related conclusion from their modelling was that the inhibition of the feedback loops themselves would likely lead to a new steady-state activity of the pathway that is not subject to negative feedback, thus enhancing the efficacy of inhibition by MEK1/2 or Raf inhibitors. This was demonstrated in a study in which SPRY2 silencing using siRNA improved the inhibition of proliferation by a Raf inhibitor [101].

In contrast to tumours with upstream activation (RTKs or Ras), those harbouring mutant B-Raf are generally sensitive to MEK1/2 or Raf inhibition. One reason for this sensitivity is that mutant B-Raf is constitutively active and insensitive to negative feedback (Fig. 2b). Hyperactivation of MEK1/2 (and ERK1/2) in B-Raf-mutated cells, therefore, continues unabated, resulting in increased transcriptional output of transforming genes with ERK1/2 being the major driver [100]. However, the induced genes include, in addition to those required for transformation, negative feedback regulators, such as DUSPs and sprouty proteins. This has two important consequences. First, negative feedback by the sprouty proteins means that Ras-GTP levels are low. This was demonstrated in B-Raf^V600E^ melanoma cells, where Ras-GTP levels could be increased by disrupting feedback via either Raf or MEK1/2 inhibition or knockdown of Spry1/2 [102]. In B-Raf^V600E^ cells, B-Raf therefore exists mostly as a monomer, which is sensitive to inhibition by Raf inhibitors [103]. In addition, the increased expression of DUSPs in B-Raf mutant cells leads to the dephosphorylation of ERK1/2 and a reduction of its apparent activity to levels that support oncogenic transformation (rather than senescence; [104, 105]). Nevertheless, due to the increased transcriptional output of these cells, several genes are induced that are essential for tumour progression. As their expression critically depends on ERK1/2 activity, those tumours are sensitive to inhibition of MEK1/2 or B-Raf. In other words, cells that evade negative feedback become sensitive to inhibition at the level of, or downstream of, the initiating mutation (i.e., B-Raf^V600E^), as those cells have lost their robustness to perturbations conferred by the negative feedback loops. This has been shown to be the case in vitro [106, 107], and a remarkably high degree of clinical efficacy has been achieved using Raf and MEK1/2 inhibitors in patients with B-Raf-mutant melanoma [108, 109]. It is, therefore, important that tumours are evaluated for their specific mutation to make an informed decision regarding the treatment options. In addition, as MEK1/2 (rather than ERK1/2) activity is a major hallmark and determinant of inhibitor selectivity (see Fig. 2), this could be used as an effective biomarker to stratify patients for treatment with either Raf, MEK1/2, or other inhibitors. Unfortunately, since Raf inhibition in normal cells expressing wild-type Raf proteins activates the ERK1/2 pathway due to relief of negative feedback and the increased formation and activation of Raf dimers, treatment with these compounds can cause ectopic ERK1/2 signalling, with cutaneous lesions (squamous cell carcinoma and/or keratoacanthoma) among the most common problems [110, 111]. However, these side effects are well manageable by surgical excision. In addition, it would be feasible to utilise combination treatments that counter the effects caused by disruption of negative feedback in healthy cells to increase the efficacy of the treatment as well as to reduce the potential side effects.

#### Negative feedback and the development of resistance to ERK1/2 pathway inhibitors

Inhibitors of B-Raf and MEK1/2 are showing substantial promise in the clinic, particularly for those tumours harbouring B-Raf mutations (see above). However, even patients who respond well to these inhibitors show signs of relapse after several months, indicating that resistance to these inhibitors has developed (adaptive resistance) [112, 113]. The main reason for resistance in tumours that are originally sensitive to B-Raf or MEK1/2 inhibitors is the re-activation of signal flux through the ERK1/2 pathway [102], resulting in increased transcription of mitogenic genes. Due to the complexity of the regulation of ERK1/2 signalling, there are many potential means by which this can be achieved. Here, we will focus on the mechanisms that are related to relief from negative feedback.

**Fig. 3:**
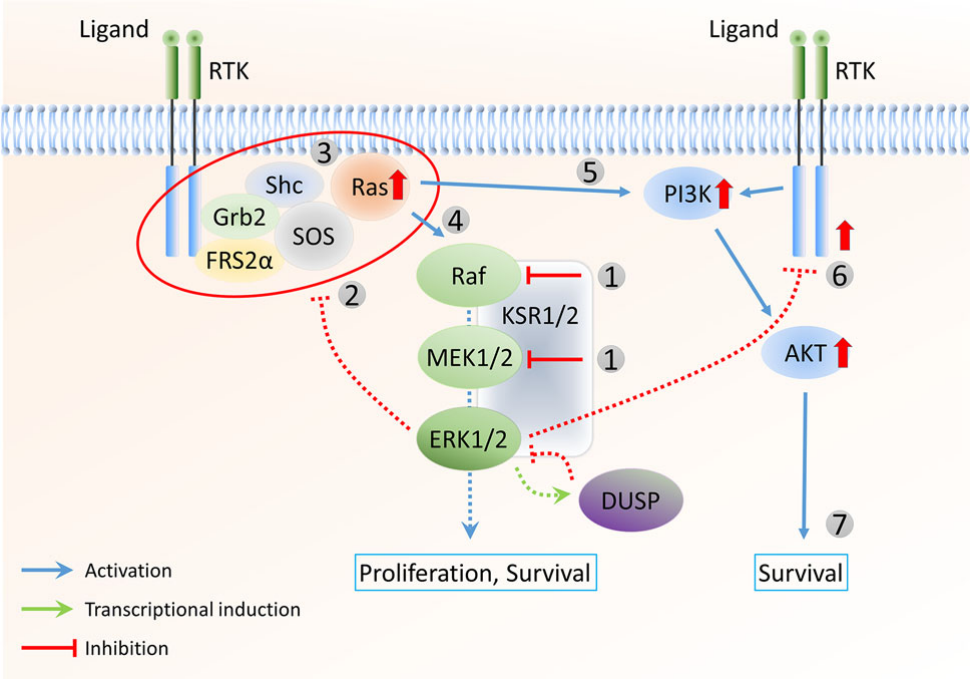
Loss of negative feedback contributes to resistance to Raf and MEK1/2 inhibitors. Raf or MEK1/2 inhibition (*1*) results in lower levels of negative feedback to upstream components (*2*). As a result, the cell is returned to an RTK signalling-competent state, where Ras and other upstream factors are able to respond to signal activation (*3*). Therefore, signal flux is increased (*4*), promoting higher ERK1/2 activity and requiring higher inhibitor doses. The increased Ras activity can also activate parallel pathways, such as the PI3K/Akt pathway (*5*). In addition, loss of negative feedback leads to the de-repression of other RTK receptors (*6*), allowing different growth factors to activate downstream signalling pathways. Activation of the PI3K/Akt pathway can promote cell survival (*7*) and reduce the dependency of the tumour on ERK1/2 signalling, likely contributing to the acquired resistance to Raf and MEK1/2 inhibitors

In B-Raf^V600E^ tumours, Ras activity is suppressed by negative feedback (Fig. 2b). Raf or MEK1/2 inhibition results in the rapid and nearly complete downregulation of ERK1/2 activity. As negative feedback partly depends on inhibitory proteins induced by ERK1/2 signalling (such as sprouty), this inhibition results in a gradual return to lower levels of negative feedback, dependent on relatively slow processes such as protein degradation. While Ras activity remains low at first because of the relatively low activity of upstream components such as RTKs, the cell is returned to a signalling-competent state, where Ras and other upstream factors are able to respond to signal activation (Fig. 3). Indeed, activation of Ras via mutation or overexpression, downregulation of the Ras negative regulator NF1, or upstream RTK overexpression and activation have been associated with resistance to Raf inhibitors in several studies [114–120]. As a result of increased Ras activation, signal flux is increased, promoting higher ERK1/2 activity and requiring higher inhibitor doses. In addition, the relief of ERK1/2-dependent feedback, together with the activation of upstream components, eventually leads to increased levels of Ras-GTP-dependent Raf dimers, which are insensitive to Raf inhibition [99]. Raf dimerisation can also result from increased expression of B-Raf or Raf-1, leading to the re-establishment of elevated ERK1/2 signalling [114, 121, 122]. Evidence for the role of Raf dimerisation in drug resistance was provided in co-immunoprecipitation experiments, which showed that MEK1/2 inhibition increased the formation of Raf-1/B-Raf dimers in melanoma cells [102]. Importantly, knockdown of Raf-1 by siRNA had no effect on basal phospho-ERK1/2 levels in B-Raf^V600E^ cells, but did reduce the extent of rebound of phospho-ERK1/2 following prolonged vemurafenib treatment. Interestingly, B-Raf mutant colon cancer cells already express significant levels of the EGFR. Inhibitors of Raf or MEK1/2, therefore, immediately upregulate EGFR activity, due to relief from negative feedback [13]. While melanoma cells require another genetic event, such as EGFR overexpression, to develop resistance, B-Raf^V600E^-positive colon cancer cells are already resistant to Raf or MEK1/2 inhibition and do not respond to treatment with vemurafenib [123].

As described above, Raf inhibition leads to an eventual steady state that is defined by a low level of negative feedback to upstream components, thus permitting Ras activation and responsiveness to RTK stimulation. Nevertheless, it follows that high flux through the pathway, once resistant to Raf inhibitors, is still sensitive to inhibition either upstream or downstream of Raf. The rationale behind this strategy is that the first drug will reduce the negative feedback in the system, increasing the sensitivity of the pathway to inhibition by the second drug. This observation led to the idea that co-targeting several points in the pathway may achieve the required near-total inhibition of ERK1/2 activity (and signal flux) and improve responses in patients with B-Raf^V600E^ melanoma [124]. Support for this idea has been gained from data showing more complete ERK1/2 inhibition and greater tumour regression in mouse xenograft models using combined Raf and MEK1/2 inhibition compared to Raf inhibition alone [102]. Promising early clinical trials suggest that these results may translate into increased therapeutic benefit [125, 126]. In addition, recent studies [103, 127] reported novel Raf inhibitors that are able to bind and inhibit both Raf monomers and dimers. These drugs may be able to address some of the resistance mechanisms, particularly in tumours with acquired resistance that is due to Raf dimerisation.

Unfortunately, the approach to specifically target ERK1/2 MAPK signalling—even with combination therapy—is not completely effective, since the relief of ERK1/2 feedback by Raf or MEK1/2 inhibition also reduces the inhibition of parallel pathways that can promote survival and reduce the dependency of the tumour on ERK1/2 signalling (Fig. 3). Several studies have shown that prolonged Raf inhibitor treatment of B-Raf^V600E^ melanoma cell lines results in the activation of the phosphatidylinositol-3-kinase (PI3K)/Akt pathway. This may occur as a result of increased activity of different RTKs, due to the release of negative feedback acting at this level. For example, Villanueva and colleagues [128] showed that, upon prolonged B-Raf inhibition, several melanoma cell lines displayed increased phosphorylation and expression of insulin-like growth factor 1 (IGF-1) receptor (IGF-1R) as well as enhanced phosphorylation of Akt. In addition, Turke et al. [10] demonstrated that MEK1/2 inhibition reduces phosphorylation of the EGFR feedback site T669, increases the activity of EGFR/ERBB3, and, consequently, activates the PI3K/Akt pathway. Rapid Akt activation by vemurafenib has also been shown in colon cancer cell lines, due to constitutive EGFR expression and the relief of negative feedback by the drug [13]. These observations present the PI3K/Akt pathway as a potential co-target alongside the ERK1/2 pathway in the treatment of B-Raf mutant melanoma and other B-Raf-driven tumours [129, 130]. Consistent with this, co-inhibition of MEK1/2 and IGF-1R or PI3K was more effective in inducing cell death in B-Raf inhibitor-resistant melanoma cells than when either inhibitor was used alone [128]. In addition, combination therapy with vemurafenib and the EGFR inhibitor panitumumab has shown promising results in early trials of B-Raf^V600E^–positive colon cancer [131] and cotreatment of colon cancer cells with vemurafenib and an EGFR (cetuximab or gefitinib) or Akt (LY294002) inhibitor was synergistic in reducing their growth [13, 132]. Another study found that tyrosine phosphorylation of the EGFR family member ERBB3 was consistently upregulated upon prolonged treatment of four melanoma cell lines with the Raf inhibitor vemurafenib [133]. Under these conditions, Akt phosphorylation was also induced, whereas incubating the cells with a monoclonal anti-ERBB3 antibody completely abrogated the increase in phosphorylation of ERBB3 receptor and Akt. Moreover, co-incubation with the anti-ERBB3 antibody was shown to potentiate the growth inhibition effects of vemurafenib in in vitro colony formation assays, inferring the possible clinical use of combining the two approaches.

Overall, these studies demonstrate the importance of understanding the mechanisms that are activated upon relief of ERK1/2-dependent negative feedback, as those efficiently promote drug resistance, particularly in B-Raf^V600E^ tumours. They also highlight the fact that multiple RTKs and signalling pathways can become activated to rescue anti-apoptotic signalling. One proposed approach to circumvent this problem is the genomic and proteomic testing of tumour samples early in and during the treatment phase [113], which would permit the identification of patient-specific mutations as well as RTK activity to inform treatment decisions. Such diagnostic tests would indicate which other pathways may need to be co-targeted, ideally before the eventual emergence of resistance.

## Final remarks

The regulation of the ERK1/2 MAPK pathway is complex and includes numerous negative feedback loops. This negative feedback has likely developed to confer robustness and effective control over this evolutionally conserved signalling pathway. While essential for the normal functioning of the cell, its ability to “rewire” signalling pathways represents a major problem for clinical intervention. Substantial progress has been made during the last few decades in understanding the complex regulation of ERK1/2 MAPK signalling. While there are still a large number of open questions, we are starting to see the benefits of applying this knowledge to targeted cancer treatment in the clinic.
